# The Laws Regulating the Bodily Temperature and the Frequency of the Pulse

**Published:** 1853-03

**Authors:** R. Lichtenfels, R. Frohlich


### ANATOMY AND PHYSIOLOGY.

*The Laws regulating the Bodily Temperature and the Frequency of the Pulse.* By R. Lichtenfels and R. Frohlich.—The authors have made a most careful series of experiments on themselves. Each experimenter is twenty-two years of age; the pulse of one of them is normally 71 per minute, that of the other 88; the normal temperature of each is 98·434. During the course of the experiments, they rose shortly before 7 A. M., took coffee between 7 and 8, had dinner at 2, and evening-coffee between 7 and 8.

1. *Daily rate of pulse and temperature.*—The influence of the period of the day, *per se*, was very trifling, but both pulse and temperature were greatly affected by food. Before the morning-coffee, the pulse was lowest; by the end of the first hour after coffee it rose, on an average of many observations, nearly 8 beats per minute; it was slightly less rapid at the end of the next hour; at the end of the third hour it was only 3·3 beats; and at the end of the fourth, 2·77 beats over the original number. The pulse did not sink to the number noted before coffee, till six hours had elapsed. The mid-day meal raised the pulse again, and this occurred apparently sooner after protein than after starchy food, but to a less extent. After the evening coffee, the pulse, which had fallen, again rose, but to a less extent, and its declension occurred more rapidly.

The temperature of the body was affected in a similar way by food, but the augmentation occurred later than the rising of the pulse; so that the temperature was often at its maximum when the pulse had fallen considerably towards the point from which it had risen. The average amount of increase is about ½ of Fah. The greatest average range of the thermometer in the course of the day (between 7 A. M. and 10 P. M.) was rather less than a degree of Fah.

2. *Influence of customary liquid.*—The experiments were performed in the afternoon; each lasted 100 minutes, and the greatest tranquillity of body was preserved. After *beer*, the pulse sank 6 or 7 beats in from 10 to 15 minutes; in 30 minutes it regained its former frequency; much before this time, the subjective feelings of slight incipient intoxication were felt. In about two hours, the pulse was heightened nearly double as much as it had been depressed. The temperature, after the use of beer, fell about one-third of a degree of Fah. After *wine*, the pulse at first fell in the same way, and then rose greatly; the temperature fell about half a degree of Fah. The same occurred with alcohol, but afterwards the temperature rose about a quarter or half a degree of Fah. Cold water lessened, at first, the number of the pulse, and lowered the temperature. In 15 minutes both returned to their former amount. Coffee, as already said, raised the pulse, but more in the morning than in the evening.

3. *Influence of fasting.*—Fasting for from 20 to 21 hours lowered both pulse and temperature. At the end, the pulse was from 12 to 16 per minute; the temperature as much as 1·8° Fah., under the normal. The curious observation (made also by Davy and Gierse) was noted, that at the period of customary meal-times both pulse and temperature slightly rose.

4. *Influence of muscular movements.*—Various experiments were tried with different kinds of movements. 1. A ten pound weight was allowed to hang from the arm for five minutes, the body being tranquil; the pulse first fell in frequency, then rose; its greatest frequency was after the termination of the experiment. When the weight was on the left arm, the rise was nearly double that which occurred when it was on the right arm. 2. A weight of one pound was held out horizontally; the pulse rose and fell remarkably several times. 3. A weight of two pounds was rapidly swung round and round with one arm, while the other was placed on a table, that the pulse might be counted. This exercise produced the greatest effect on the pulse, raising it sometimes from 30 to 50 beats. 4. Long-continued moderate exercise, carried on to fatigue, raised the pulse greatly for some considerable time, but never produced the enormous rise noted in the previous kind (3) of muscular exertion.

5. *Influence of narcotic poisons.*—*Belladonna* and *atropine* at first diminished the frequency of the pulse (16 to 20 beats), but after a variable time (50 to 117 minutes,) the pulse again rose (12 to 30 beats.) The smaller doses produced greater primary sinking than the large, but required much longer time to do so; on the contrary, the larger doses produced much greater secondary rising; that is to say, the maximum sinking-point is inversely, and the maximum rising-point is directly, proportioned to the amount of the drug. It might be said that small doses depress, larger excite, the pulse. The temperature was diminished in all cases. *Opium*, especially in small doses, caused rising of the pulse, but afterwards there was great sinking, and the temperature diminished. The *Cannabis Indica* produced many periods of rising and falling; the temperature rose for about four hours, and to as great a degree as ·7° or 1° Fah. *Chloroform* and *ether*, if not pushed to too deep narcotism, raised both temperature and pulse.—*British and Foreign Medico-Chirurgical Review.*

